# Liraglutide-Induced Weight Loss May be Affected by Autonomic Regulation in Type 1 Diabetes

**DOI:** 10.3389/fendo.2019.00242

**Published:** 2019-04-12

**Authors:** Christian Stevns Hansen, Christian Seerup Frandsen, Jesper Fleischer, Dorte Vistisen, Jens Juul Holst, Lise Tarnow, Filip Krag Knop, Sten Madsbad, Henrik Ullits Andersen, Thomas Fremming Dejgaard

**Affiliations:** ^1^Steno Diabetes Center Copenhagen, Gentofte, Denmark; ^2^Department of Endocrinology, Hvidovre Hospital, University of Copenhagen, Hvidovre, Denmark; ^3^Clinical Institute of Medicine, Aarhus University, Århus, Denmark; ^4^Department of Biomedical Sciences, Faculty of Health and Medical Sciences, University of Copenhagen, Copenhagen, Denmark; ^5^Faculty of Health and Medical Sciences, Novo Nordisk Foundation Center for Basic Metabolic Research, University of Copenhagen, Copenhagen, Denmark; ^6^Nordsjællands Hospital Hillerød, University of Copenhagen, Hillerød, Denmark; ^7^Clinical Metabolic Physiology, Steno Diabetes Center Copenhagen, Gentofte Hospital, University of Copenhagen, Hellerup, Denmark; ^8^Department of Clinical Medicine, Faculty of Health and Medical Sciences, University of Copenhagen, Copenhagen, Denmark

**Keywords:** liraglutide, autonomic neuropathy, weight loss, insulin requirements, type 1 diabetes

## Abstract

The role of the autonomic nervous system in the efficacy of glucagon-like peptide-1 receptor agonists (GLP-1 RA) in patients with type 1 diabetes is unknown. We assessed the association between autonomic function and weight loss induced by the GLP-1 RA liraglutide.

**Methods:** Lira-1 was a randomized, double-blind, placebo-controlled trial assessing the efficacy and safety of 1.8 mg liraglutide once-daily for 24 weeks in overweight patients with type 1 diabetes. Autonomic function was assessed by heart rate response to deep breathing (E/I ratio), to standing (30/15 ratio), to the Valsalva maneuver and resting heart rate variability (HRV) indices. Associations between baseline the cardiovascular autonomic neuropathy (CAN) diagnosis (> 1 pathological non-resting test) and levels of test outcomes on liraglutide-induced weight loss was assessed by linear regression models.

**Results:** Ninety-nine patients with mean age 48 (SD 12) years, HbA_1c_ 70 (IQR 66;75) mmol/mol and BMI of 30 (SD 3) kg/m^2^ were assigned to liraglutide (*N* = 50) or placebo (*N* = 49). The CAN diagnosis was not associated with weight loss. A 50% higher baseline level of the 30/15 ratio was associated with a larger weight reduction by liraglutide of −2.65 kg during the trial (95% CI: −4.60; −0.69; *P* = 0.009). Similar significant associations were found for several HRV indices.

**Conclusions:** The overall CAN diagnosis was not associated with liraglutide-induced weight loss in overweight patients with type 1 diabetes. Assessed separately, better outcomes for several CAN measures were associated with higher weight loss, indicating that autonomic involvement in liraglutide-induced weight loss may exist.

## Introduction

Glucagon-like peptide-1 (GLP-1) is a gut-derived hormone with anorexigenic properties (1). The GLP-1 receptor agonist (GLP-1RA) liraglutide is known to induce weight loss in patients with type 2 diabetes as well as in patients with type 1 diabetes (2–5). However, the exact mechanisms by which GLP-1 exerts its anorectic effects are not fully clarified. Activation of GLP-1 receptors in peripheral vagal neurons (6, 7) seems to be involved, suggesting that dysfunction of the vagal nerve may affect the body weight-reducing effect of liraglutide in patients. If this is the case, a substantial subset of people with diabetes may experience a reduced effect of treatment as autonomic neuropathy is a common complication to diabetes. Prevalence rates of cardiovascular autonomic neuropathy (CAN) in people with type 1 diabetes and type 2 diabetes, respectively, range from 20% in unselected diabetes populations (8, 9) to 65% in patients with long-standing diabetes(10). We hypothesize that autonomic dysfunction might be expected to influence the efficacy of GLP-1RAs in type 1 patients. Here, we explored the possible association between CAN measures and liraglutide-induced weight loss, insulin requirements and gastric emptying rate in patients with type 1 diabetes.

## Participants and Methods

### Study Design

The present study is a secondary analysis of data from the Lira-1 study (3). Lira-1 was a single-center, parallel-group, double-blinded, randomized, placebo-controlled trial performed at Steno Diabetes Center (Gentofte, Denmark). In total, 100 overweight (BMI> 25 kg/m^2^) patients with type 1 diabetes and insufficient glycaemic control (HbA1c > 64 mmol/mol (8%)) were randomly allocated (1:1) to receive 24 weeks of identical liraglutide 1.8 mg once daily (QD) (Novo Nordisk, Måløv, Denmark) or placebo QD (saline injection), as an add-on to existing insulin treatment. Liraglutide dose was successively increased weekly by 0.6 mg from 0.6 mg QD to 1.8 mg QD. To reduce the risk of hypoglycaemia bolus and basal insulin doses were reduced by 33 and 25% at randomization. Insulin doses were adjusted throughout the trial aiming at preprandial glucose targets of 4–7 mmol/L and a postprandial glucose of <10 mmol/L.

The study included 5 visits at weeks 0, 3, 12, 23, and 24 at which information about concomitant medication, basal and bolus insulin doses, bodyweight, blood pressure, and heart rate were collected. Blood and urine samples were collected at these visits. Details about the study design and results regarding main outcomes have been described previously (3). The study was approved by the Scientific Ethical Committee of the Capital Region of Denmark (H-1-2012-031), the Danish Medicines Authority (EudraCT: 2012-001150-26), and the Danish Data Protection Agency. The trial is registered with ClinicalTrials.gov, number NCT01612468.

### Assessment of Autonomic Function

Autonomic function was assessed by measures of CAN; heart rate variability (HRV) and cardiovascular autonomic reflex tests (CARTs). Assessments were done at baseline (week 0) and at end of trial (week 24) by 5-min of passive supine recordings of HRV followed by three measures of CARTs.

HRV indices were analyzed using normal statistical description (time domain) and by estimating frequency-specific fluctuations in HRV (frequency domain) (11). HRV indices in the time domain were calculated by the root mean square of the sum of the squares of differences between consecutive R–R intervals (RMSSD) and as the standard deviation of normal-to-normal intervals (SDNN). In the frequency domain, the calculations of power were done in the low-frequency (LF) power (0.04–0.15 Hz) band, the high-frequency (HF) power (0.15–0.4 Hz) band and in the total frequency (≤ 0.4 Hz) spectrum autoregressive model. RMSSD and HF are measures of parasympathetic activity and SDNN, LF, total power and LF/HF-ratio are measures of both the parasympathetic and sympathetic activity (11).

The three standard CARTs recommended for diagnosing CAN (12) were performed: the lying-to-standing test (30/15) testing a mix of the sympathetic and the parasympathetic nervous system, the deep breathing test (E/I ratio) primarily testing parasympathetic nervous system and the Valsalva maneuver primarily testing sympathetic nervous system. CARTs were performed in the mentioned order and in accordance with procedures suggested by Ewing (13). CAN assessment was performed after 5 min of supine resting in a quiet room at 18–23 degrees Celsius. Patients were fasting and refrained from strenuous physical activity 24 h prior to the examination. Smoking was not allowed 3 h prior to testing.

All CARTs and HRV measures were analyzed as continuous variables. Age-dependent cut-off levels as recommended by Spallone et al. (14) were used to define pathological results of CARTs. Manifest CAN diagnosis was defined as having more than one pathological CARTs as recommended by the American Diabetes Association (15). Higher values of the all autonomic measures imply better autonomic function.

Resting HRV indices and CARTs were recorded by trained technicians using a Vagus^tm^ device (Medicus Engineering, Aarhus, Denmark). The Vagus device is a handheld device that enables the measurement of R-R intervals of a two-point electrocardiogram. Patients connects to the device by holding on to two electrodes at each end. Measure are obtained automatically. Instructions of breathing frequency, when to rise and when to blow though a mouthpiece at 40 mmHg are given on a display on the device (16).

### Assessment of Gastric Emptying Rate

The first 40 patients included in the Lira-1 trial were subjected to a standardized liquid mixed meal test (Nutridrink Protein (Nutricia, Schiphol, Netherlands); 200 mL containing 300 kcal, 31,2 g carbohydrate, 20 g protein, and 10,6 g fat) at week 0, 3 and 24. Gastric emptying rate was assessed by the paracetamol absorption test (17).

### Statistical Methods

Patient characteristics are presented as means with standard deviations (SD), as medians with interquartile range (IQR) for characteristics with a skewed distribution or as percentages.

Outcomes were repeated measurements of body weight in kilograms, daily insulin requirements (units/day) andgastric emptying assessed by the paracetamol absorption test. CAN measures as continuous variables and the CAN diagnosis as a binary variable were used as determinants. All analyses including HRV were adjusted for 5 min resting heart rate at the time of testing. Analyses of HRV were also performed without adjustments for heart rate to assess the effect of heart rate on estimates. The trapezoidal rule was used to calculate AUC_0−240min_ during the meal test (3).

Associations were modeled by linear mixed-effect models with a patient-specific random intercept to account for the correlation of repeated measurements within patients. We tested for a modifying effect of having the CAN diagnosis at baseline or not on liraglutide-induced changes in outcomes. Also, the modifying effect of increasing (better) levels of CAN measures at baseline on the effect of liraglutide-induced changes in outcomes was tested. All analyses were performed as an intention to treat analysis. To fulfill the requirement of a normal distribution of the model residuals, all determinants were log_1.5_-transformed and the following outcomes were log-transformed: total insulin requirements per day and measures of gastric emptying. For log-transformed outcomes, estimates are presented as % change due to subsequent back transformation of results in log-scale. Estimates are therefore a function of a 50% higher level of determinants at baseline on outcomes, e.g. liraglutide-induced weight loss. An increase of 50% was chosen to assess a clinically relevant difference in autonomic measures. Where relevant, analyses of a 50% higher change in determinants during the trial period were assessed. Standardized regression coefficients for log_1.5_-transformed CARTs and HRV measures were further calculated to allow for direct comparison between the parameter estimates.

Group differences (liraglutide vs. placebo) in change in continuous CAN measures between baseline and end of trial were assessed by linear regression analyses adjusting for baseline values of the CAN measure analyzed.

Statistical significance was inferred at a two-tailed *P*-value < 0.05.

Analyses were performed using SAS version 9.4 (SAS Institute, Cary, NC) and R version 3.2.1 (The R Foundation for Statistical Computing, www.R-project.org).

## Results

Of the 100 patients enrolled in the Lira-1 trial, one patient in the placebo group had no usable CAN measures, leaving 50 patients in the liraglutide and 49 patients in the placebo arm for analysis. During the trial four patients assigned to liraglutide treatment and six patients in the placebo arm discontinued the trial.

Patients were predominantly male (60% in the liraglutide group and 67.3% in the placebo group) with a mean age of 48 (SD 12) years, a median HbA_1c_ of 70 (IQR 66;75) mmol/mol and a mean BMI of 30 (SD 3.5) kg/m^2^. Baseline demographic, anthropometric and cardio-metabolic markers were similar in the two groups except for diabetes duration which on average was 4 years longer in the placebo group. At baseline, 15 patients (30%) in the liraglutide group and 12 patients (25%) in the placebo group had the CAN diagnosis. CARTs and HRV measures were similar in the two groups (Table 1).

**Table 1:** Baseline characteristics by treatment group.

	**Liraglutide (*N* = 50)**	**Placebo (*N* = 49)**
Sex (male), (N/%)	30 (60.0)	33 (67.3)
Age (years)	47.7 (13.3)	48.9 (11.7)
HbA_1c_ (mmol/mol)	70 (66;74)	70 (66;76)
HbA_1c_ (%)	8.6 (8.2;8.9)	8.6 (8.2;9.1)
Bodyweight (kg)	92.4 (14.5)	92.9 (13.0)
Body mass index (kg/m^2^)	30.2 (3.5)	29.9 (3.4)
Diabetes duration (years)	17 (11;24)	21 (16;34)
Insulin dose per kilo per day	0.6 (0.2)	0.6 (0.2)
Total cholesterol (mmol/L)	4.4 (4;5)	4.5 (4;5.1)
HDL cholesterol (mmol/L)	1.3 (0.4)	1.3 (0.3)
LDL cholesterol (mmol/L)	2.7 (0.9)	2.8 (0.9)
Systolic blood pressure (mmHg)	131 (16)	131 (16)
Diastolic blood pressure (mmHg)	82 (9)	81 (7)
Beta blocker (N/%)	0.0 (0)	4 (8.2)
Diuretics (N/%)	11 (22.0)	15 (30.6)
ACE inhibitor (N/%)	13 (26.0)	19 (38.8)
ARBs (N/%)	6 (12.0)	10 (20.4)
CAN diagnosis (N/%)	15 (30.0)	12 (24.5)
Early CAN (N/%)	16 (33.3)	18 (37.5)
Pathological E/I ratio (N/%)	22 (44.0)	22 (44.9)
Pathological 30/15 ratio (N/%)	12 (24.0)	11 (22.4)
Pathological Valsalva (N/%)	16 (33.3)	13 (27.1)
E/I ratio	1.2 (1.1;1.3)	1.2 (1.1;1.3)
30/15 ratio	1.1 (1;1.3)	1.1 (1.1;1.3)
Valsalva	1.4 (1.2;1.6)	1.4 (1.2;1.7)
SDNN (ms)	28.4 (17.1;43.3)	25.7 (19.9;32.7)
RMSSD (ms)	17.9 (9.0;29.2)	14.3 (9.7;23.6)
High frequency power (ms^2^)	31.8 (8.8;117.3)	27.6 (9.1;72.2)
Low frequency power (ms^2^)	86.2 (19.0;188.1)	58.2 (25.6;100.4)
Total power (ms^2^)	220.3 (94.2;576.3)	197.4 (132.5;321.8)
LF/HF ratio	2.6 (1.6;5.4)	2.3 (1.2;3.7)
Heart rate (beats/minute)	72.0 (11.5)	69.9 (10.3)

*Data are in means with standard deviation (SD) in brackets or in medians with interquartile range (IQR) in brackets or numbers (n) with percent in brackets. HDL, high-density lipoprotein; LDL, low-density lipoprotein ACE, angiotensin-converting-enzyme inhibitor; ARBs, angiotensin II receptor blockers; CAN, cardiovascular autonomic neuropathy; RMSSD, the root mean square of the sum of the squares of differences between consecutive R–R intervals; SDNN, standard deviation of normal-to-normal intervals; LF/HF-ratio, Low frequency power / High frequency power ratio*.

### Weight Loss

In the liraglutide group, patients with and without CAN had similar weight change of −6.08 kg (95% CI −11.76;-0.38) vs. −5.77 kg (95% CI −13.51;1.98), respectively (*P* = 0.438 for between-group difference).

The effect of the CAN diagnosis at baseline on weight loss were similar in the liraglutide group and the placebo group (*P* = 0.513 for group difference).

In the liraglutide group a 50% higher baseline 30/15 ratio was associated with a larger weight change of −2.65 kg (95% CI −4.60; −0.69 *P* = 0.009). Similar numerical results were found for the E/I ratio and the Valsalva maneuver and liraglutide induced weight loss but did not reach statistical significance, no differences between groups were observed (Table 2).

**Table 2:** The association between CAN measure and weight change (measured in kilo from baseline to follow-up).

	**Liraglutide**	**Placebo**	***P* for group difference**
**CARTS**			
E/I ratio	−0.01(−1.95;1.94)[0.994]	2.36(0.08;4.64)[0.041]	0.121
30/15 ratio	−2.65(−4.60;−0.69)[0.009]	1.32(−0.64;3.27)[0.189]	0.005
Valsalva	−0.91(−2.36;0.45)[0.190]	1.04(−0.35;2.43)[0.144]	0.050
**HEART RATE VARIABILITY INDICES**			
SDNN (ms)	−0.48(−0.93;−0.04)[0.032]	0.65(0.07;1.22) [0.028]	0.023
RMSSD (ms)	−0.31(−0.69;0.06)[0.105]	0.15(−0.29;0.59) [0.504]	0.120
High frequency power (ms^2^)	−0.11(−0.28;0.05)[0.179]	0.02(−0.18;0.22) [0.833]	0.302
Low frequency power (ms^2^)	−0.24(−042;−0.06)[0.010]	0.23(−0.01;0.48) [0.066]	0.003
Total power (ms^2^)	−0.24(−0.46;−0.01)[0.039]	0.30(0.03;0.58) [0.031]	0.003
LF/HF ratio	−0.19(−0.48;0.06)[0.147]	0.19(−0.05;0.044)[0.127]	0.035
**HEARTS RATE**			
Heart rate (beats/minute)	1.41(−0.30;3.13)[0.107]	0.47(−1.40;2.33)[0.623]	0.464

*Results are weight change in kilo from baseline to follow-up with 95% CI as a result of a 50% higher level of CAN measures at baseline [P-value]. CARTS, cardiovascular autonomic reflex tests; E/I ratio, heart rate response to deep breathing; 30/15 ratio, heart rate response to standing; SDNN, standard deviation of normal-to-normal intervals; RMSSD, the root mean square of the sum of the squares of differences between consecutive R–R intervals; LF, low-frequency; HF, high-frequency*.

**Figure 1:**
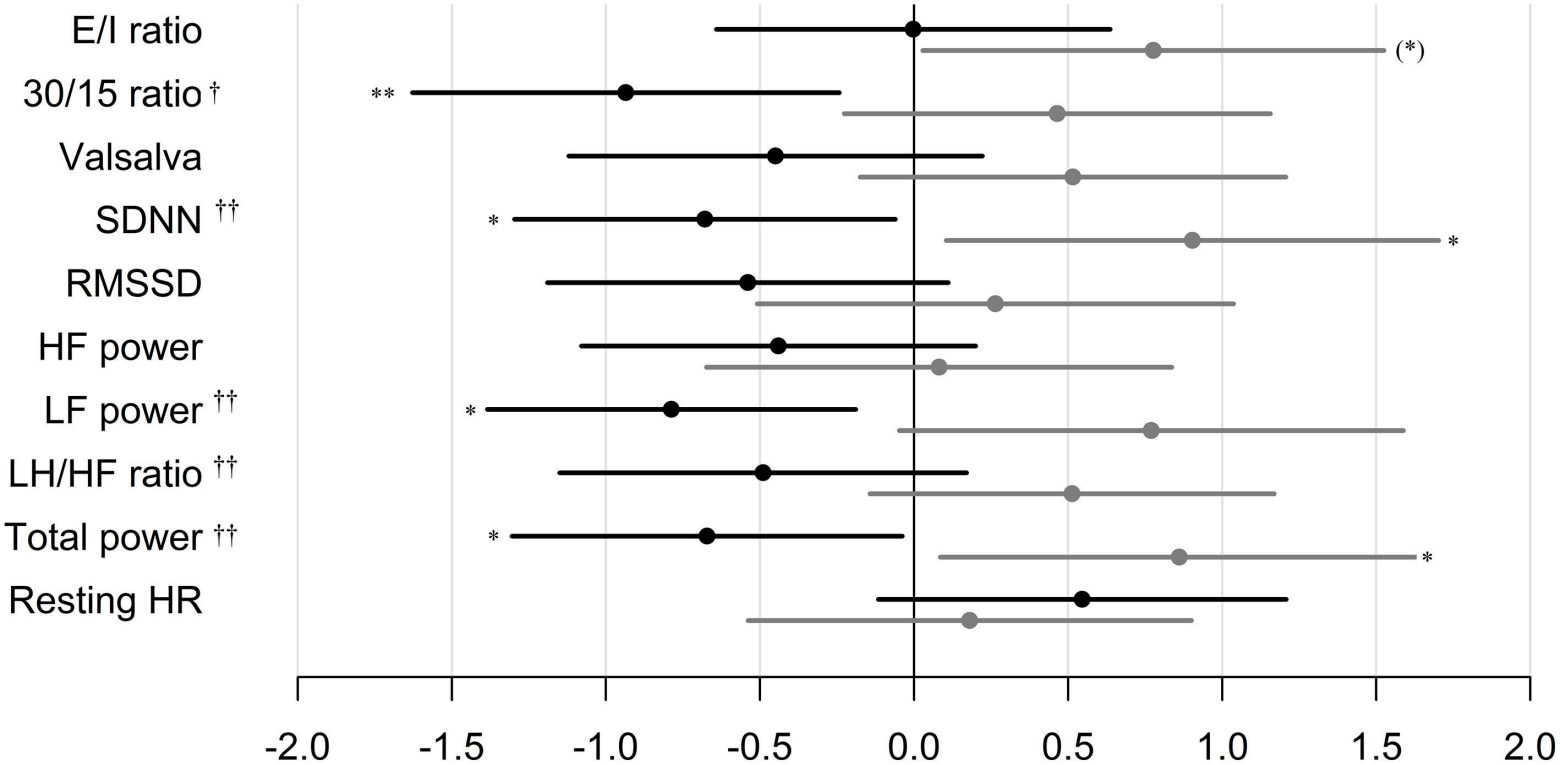
Standardized regression coefficients with 95% CL of the associations between CAN measures and weight change during trial. Estimates are in kilo on the log-scale by one SD increase in log1.5 of the determinant. Black: Liraglutide group. Gray: placebo group. E/I ratio, heart rate response to deep breathing; 30/15 ratio, heart rate response to standing; SDNN, standard deviation of normal-to-normal intervals; RMSSD, the root mean square of the sum of the squares of differences between consecutive R–R intervals; LF, low-frequency; HF, high-frequency. *P*-value for between group difference indicated by † = <0.01, †† = <0.005, *P*-value for change within group indicated by *P < 0.05, ** *P* < 0.01. * in brackets indicate no group difference.

In patients treated with liraglutide, higher baseline HRV indices SDNN, LF, and total power were significantly associated with larger weight loss during the trial. For the remaining HRV indices a similar but non-significant association to liraglutide-induced weights loss was seen. In the placebo group, higher baseline SDNN and total power were associated with body weight gain during trial. Resting heart rate was not associated with body weight change in any of the groups (Table 2). As illustrated in Figure 1, increasing levels of CAN measures associated with weight change had similar effects on weight change elicited by liraglutide when assessed in standardized regression models.

In the placebo group, patients with and without CAN had similar weight change of −1.05 kg (95% CI −8.30; 6.20) vs. 0.67 kg (95% CI −4.10; 5.44), respectively (*P* = 0.0993 for between-group difference).

### Insulin Requirements

**Figure 2:**
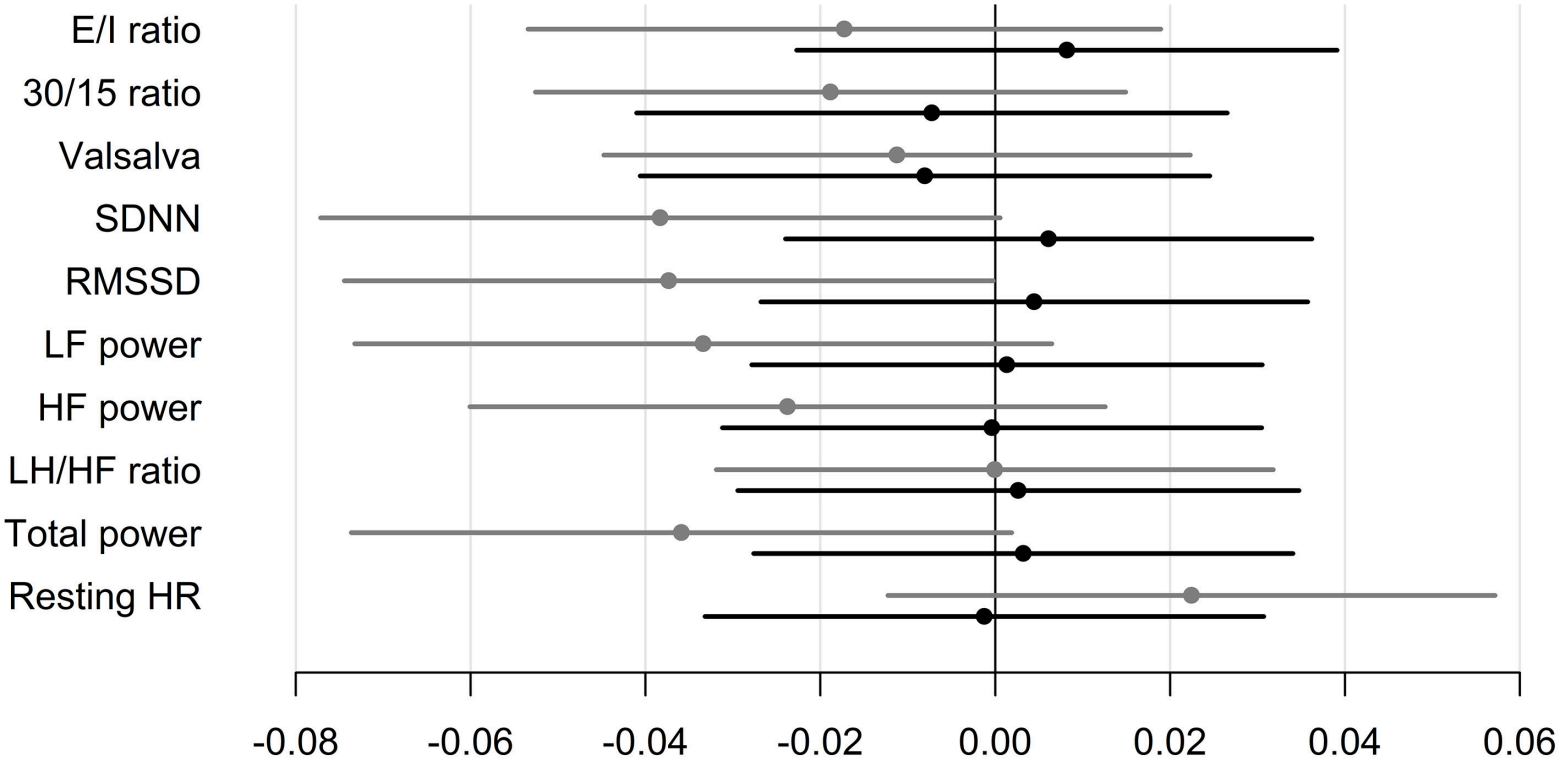
Standardized regression coefficients with 95% CL of the associations between CAN measures and change in insulin requirements during trial. Estimates are in IU per day on the log-scale by one SD increase in log1.5 of the determinant. Black: Liraglutide group. Gray: placebo group. E/I ratio, heart rate response to deep breathing; 30/15 ratio, heart rate response to standing; SDNN, standard deviation of normal-to-normal intervals; RMSSD, the root mean square of the sum of the squares of differences between consecutive R–R intervals; LF, low-frequency; HF, high-frequency.

CAN diagnosis was not associated with changes in insulin requirements in any of the groups. Neither, CARTs, HRV indices or resting heart rate were associated with changes in insulin requirements in any of the group (Table 3 and Figure 2).

**Table 3:** The association between CAN measures and change in insulin use (in percentage) during trial.

	**Liraglutide**	**Placebo**	**P for group difference**
**CARTS**			
E/I ratio	−2.52(−6.67;12.63)[0.604]	−5.11(−15.00;5.92)[0.351]	0.293
30/15 ratio	−2.02(−10.95;7.79)[0.675]	−5.19(−13.82;4.31)[0.275]	0.633
Valsalva	−1.60(−7.86;5.07)[0.629]	−2.24(−8.62;4.59)[0.512]	0.629
**HEART RATE VARIABILITY INDICES**			
SDNN (ms)	0.44(−1.70;2.62)[0.691]	−2.70(−5.37;0.04)[0.054]	0.076
RMSSD (ms)	0.25(−1.51;2.06)[0.780]	−2.10(−4.15;−0.01)[0.050]	0.091
High frequency power (ms^2^)	−0.01(−0.81;0.80)[0.981]	−0.62(−1.56;0.33)[0.201]	0.334
Low frequency power (ms^2^)	0.04(−0.84;0.93)[0.928]	−1.01(−2.20;0.20)[0.102]	0.167
LF/HF ratio	0.10(−1.11;1.32)[0.872]	0.00(−1.20;1.21)[0.997]	0.906
Total power (ms^2^)	0.11(−0.97;1.20)[0.839]	−1.25(−2.55;0.07)[0.04]	0.115
**HEARTS RATE**			
Heart rate (beats/minute)	−0.32(−8.25;8.29)[0.940]	5.99(−3.13;15.97)[0.206]	0.323

*Results are weight change in kilo from baseline to follow-up with 95% CI as a result of a 50% higher level of CAN measures at baseline [P-value]. CARTS, cardiovascular autonomic reflex tests; E/I ratio, heart rate response to deep breathing; 30/15 ratio, heart rate response to standing; SDNN, standard deviation of normal-to-normal intervals; RMSSD, the root mean square of the sum of the squares of differences between consecutive R–R intervals; LF, low-frequency; HF, high-frequency*.

### Gastric Emptying

In the subset of 40 patients who underwent liquid mixed meal testing, liraglutide induced a delay in gastric emptying rate measured by paracetamol as reported earlier (3). The CAN diagnosis at baseline did not affect the change in gastric emptying rate induced by liraglutide when assessed by total AUC_0−240min_ (*P* = 0.406).

In the liraglutide group a 50% higher level of E/I ratio at baseline was associated with lower effect of liraglutide on paracetamol AUC_0−240min_ of −15.10 mmol/L x min (95% CI −26.78; −1.56 *P* = 0.034). No other baseline autonomic measures were associated with gastric emptying (Table 4).

**Table 4:** CAN measures and association to gastric emptying assessed by AUC_0−240min_ of serum paracetamol.

	**Liraglutide**	**Placebo**	***P* for group difference**
**CARTS**			
E/I ratio	−15.10(−26.78;−1.56)[0.034]	11.03(−7.80;33.70)[0.274]	0.028
30/15 ratio	16.39(−3.53;40.43)[0.118]	12.04(−7.43;35.60)[0.247]	0.778
Valsalva	11.23(−1.40;25.48)[0.089]	−4.28(−6.49;16.29)[0.454]	0.433
**HEART RATE VARIABILITY INDICES**			
SDNN (ms)	0.50(−3.34;4.49)[0.802]	1.34(−3.94;6.92)[0.627]	0.804
RMSSD (ms)	1.75(−1.56;5.17)[0.308]	−0.23(−4.96;5.70)[0.933]	0.636
High frequency power (ms^2^)	0.49(−1.07;2.07)[0.541]	−0.61(−2.64;1.46)[0.564]	0.401
Low frequency power (ms^2^)	0.00(−1.28;1.30)[0.998]	1.08(−1.56;3.79)[0.429]	0.474
Total power (ms^2^)	0.00(−1.79;1.83)[0.998]	−0.08(−2.68;2.60)[0.956]	0.962
LF/HF ratio	−0.67(−2.51;1.20)[0.481]	1.14(−0.85;3.17)[0.269]	0.193
**HEARTS RATE**			
Heart rate (beats/minute)	−13.94(−24.23;−2.25)[0.024]	−5.31(−17.79;9.07)[0.453]	0.322

*Results are percentage change in gastric emptying assessed by AUCtotal 0–240 min paracetamol absorption testfrom baseline to follow-up with 95% CI as a result of a 50% higher level of CAN measures at baseline. CARTS, cardiovascular autonomic reflex tests; E/I ratio, heart rate response to deep breathing; 30/15 ratio, heart rate response to standing; SDNN, standard deviation of normal-to-normal intervals; RMSSD, the root mean square of the sum of the squares of differences between consecutive R–R intervals; LF, low-frequency; HF, high-frequency*.

A 50% increase in paracetamol AUC_0−240min_ during trial was associated with a decrease in body weight of 5.8% (95% CI 1.4; 10.0 *P* = 0.016) in the liraglutide group.

### CAN Measures

Liraglutide treatment did not elicit changes in CAN measures from baseline to end of trial, but increased resting heart rate by 8 beats per min. compared to placebo as described previously (3). A study effect on the 30/15 ratio was seen as a decrease from baseline to follow-up of 0.06 (95% CI −0.11;0.02, *P* = 0.004 in the lira group and P = 0.007 in the placebo group) in both treatment groups, with no between-group difference (*P* = 0.900). All other measures of CAN and resting heart rate remained unchanged in the placebo group throughout the trial (Table 5).

**Table 5:** Treatment effects on CAN outcomes.

	**Baseline**		**Week 24**		**Change from baseline to week 24**		
	**Liraglutide**	**Placebo**	**Liraglutide**	**Placebo**	**Liraglutide**	**Placebo**	**P for group difference**
**CARTS**							
E/I ratio	1.2 (1.1;1.3)	1.2 (1.1;1.3)	1.2 (1.1;1.3)	1.1 (1.1;1.3)	−0.01(−0.03;0.02)[0.547]	0.00(−0.03;0.03)[0.875]	0.757
30/15 ratio	1.1 (1;1.3)	1.1 (1.1;1.3)	1.1 (1;1.2)	1.1 (1;1.2)	−0.06(−0.11;−0.02)[0.004]	−0.06(−0.11;−0.02)[0.007]	0.897
Valsalva	1.4 (1.2;1.6)	1.4 (1.2;1.7)	1.4 (1.2;1.6)	1.4 (1.2;1.7)	−0.05(−0.13;0.03)[0.250]	−0.01(−0.10;0.08)[0.799]	0.527
**HEART RATE VARIABILITY INDICES**							
SDNN (ms)	28.4 (17.1;43.3)	25.7 (19.9;32.7)	22.7 (13.9;34.7)	23.2 (16.4;36.9)	4.90(−8.71;18.51)[0.481]	−0.27(−15.05;14.51)[0.971]	0.576
RMSSD (ms)	17.9 (9;29.2)	14.3 (9.7;23.6)	12.7 (8.5;20.7)	14.8 (8.6;23.7)	10.10(−6.71;29.92)[0.239]	−1.73(−19.98;16.53)[0.853]	0.350
High frequency power (ms^2^)	31.8 (8.8;117.3)	27.6 (9.1;72.2)	23.2 (6.5;79.9)	24.7 (6.5;61.2)	757.92(−278.38;1803.22)[0.155]	−28.2(−1163.4;1107.0)[0.961]	0.322
Low frequency power (ms^2^)	86.2 (19;188.1)	58.2 (25.6;100.4)	74.7 (35.8;258.9)	57.2 (22.8;107.4)	1083.35(−433.09;2599.79)[0.161]	9.60(−1656.52;1637.32)[0.991]	0.339
LF/HF ratio	2.6 (1.6;5.4)	2.3 (1.2;3.7)	3 (1.1;7.2)	2.7 (1.2;5.7)	0.31(−1.11;1.74)[0.668]	0.66(−0.88;2.21)[0.402]	0.953
Total power (ms^2^)	220.3 (94.2;576.3)	197.4 (132.5;321.8)	173.5 (72.5;460.2)	134.2 (78.9;356.1)	2542.76(−999.15;6084.67)[0.159]	−31.83(−3878.49;3814.84)[0.987]	0.334
**HEARTS RATE**							
Heart rate (beats/minute)	72 (11.5)	69.9 (10.3)	76.9 (11.7)	70.9 (9)	4.68(1.99;7.36)[0.001]	0.18(−2.74;3.10)[0.903]	0.006

*Absolute values of CAN outcomes at baseline and at end of trial in the two groups are shown medians with interquartile range (IQR) in brackets. Change in CAN outcomes during trial are shown as absolute values with 95% CI in brackets and P-value for test of difference in square brackets. P-value for test of between group differences in change in CAN measures from baseline to end of trial are shown in square brackets. CARTs, cardiovascular autonomic reflex tests E/I ratio, heart rate response to deep breathing; 30/15 ratio, heart rate response to standing; E/I ratio, heart rate response to deep breathing; 30/15 ratio, heart rate response to standing; RMSSD, the root mean square of the sum of the squares of differences between consecutive R–R intervals, SDNN, standard deviation of normal-to-normal intervals, LF/HF-ratio, Low frequency power / High frequency power ratio*.

## Discussion

The CAN diagnosis *per se* was not associated with liraglutide induced weight loss. This could be attributed to the composite nature of the CAN diagnosis which is comprised by a diverse array of autonomic measures, limiting the ability to assess the individual components of the autonomic nervous system (18). On the other hand, our findings may suggest that autonomic function is associated with liraglutide-induced weight loss in overweight patients with type 1 diabetes and insufficient glycaemic control. We found that higher baseline values of mixed parasympathetic and sympathetic measures (the 30/15 ratio and the HRV indices SDNN, LF power and total power) were associated with increased liraglutide induced weight loss. Specific parasympathetic measures were not significantly associated with weight loss in the liraglutide group, which indicates that the modifying effect of autonomic function on liraglutide induced weight loss is not solely mediated by vagal function, but rather by a complex interplay between the sympathetic and parasympathetic nervous system.

The lack of association between the CAN diagnosis and liraglutide-induced weight loss may indicate the activation of peripheral autonomic nerves is not a prerequisite for GLP-1 RA-induced weight-loss. Direct stimulation of the central nervous system may be required for weight loss.

Native GLP-1 and liraglutide may not directly pass the blood brain barrier, but may access certain regions of the brain via the circumventricular organs (19–22), and it has been suggested that exogenous GLP-1 may in this way have a direct effect on receptors in the central nervous system and thereby induce weight loss by increased satiety (23, 24). In mice, liraglutide effects on food intake were abolished after genetic deletion of brain receptors, but remained after deletion of peripheral autonomic nerves (25). In summary, our results do not rule out that the peripheral autonomic nervous system plays a role in liraglutide induced weight loss.

A potential cause of liraglutide induced weight loss could be the reduction in insulin use seen in the liraglutide group (3). However, autonomic function was not associated with insulin requirements during trial.

Only baseline E/I ratio was associated with liraglutide induced deceleration of gastric emptying, indicating that vagal nerve function might be associated with the effect of liraglutide on gastric motility. Earlier studies have shown that GLP-1-induced deceleration of gastric emptying is lost after vagotomy in non-diabetic individuals (26) suggesting that the effect of GLP-1 on gastric emptying is mediated via the vagus nerve as indicated by our findings. Presently, however it remains controversial whether liraglutide's effect on gastric emptying contributes to liraglutide-induced weight loss (27, 28). As the baseline E/I ratio was not associated with weight loss, and as no other CAN measure were associated with gastric emptying, we conclude that the association between baseline CAN measures and liraglutide induced weight loss was not mediated through gastric emptying rate.

As CAN measures were not affected by liraglutide or placebo treatment in this trial it is unlikely that associations between baseline measures of CAN and study outcomes are a result of changes in autonomic nervous function. The E/I ratio did decline in both study arms at comparable magnitudes which could be attributed to an overall study effect. In contrast, a recent study in type 2 diabetes patients reported that 12 weeks of liraglutide treatment induced detrimental reductions in several HRV indices (29). However, 18 months of treatment with the short-acting GLP1-RA exenatide had no effect on CAN measures in patients with type 2 diabetes (30) which is in line with the findings of the present study.

As the study was not designed to assess the association between CAN and the efficacy of liraglutide it may be underpowered to show significant association on more CAN parameters than presented here.

Measures of autonomic function used in the present study are not purely associated to either branch of the autonomic nervous system, which hampers the ability to conclude on associations specifically related to either the sympathetic or the parasympathetic nervous system. Measures of blood pressure changes as a response to Valsalva maneuver and active standing could have enabled a more specific assessment of sympathetic nervous function.

The present study is a *post-hoc* analyses of data from a randomized controlled trial designed to estimate the efficacy of liraglutide in overweight patients with type 1 diabetes. This study was not designed or powered to assess differences in efficacy in patients with or without autonomic dysfunction. Hence, the lack of a modifying effect of the CAN diagnosis on the efficacy of liraglutide-induced weight change may be due to lack of power. Confirmatory studies where participants are stratified by CAN status must be performed before conclusions can be made on whether autonomic neuropathy affects the weight-lowering effect of GLP-1 RAs.

In conclusion, our results suggest that weight loss induced by liraglutide in type 1 diabetes patients is not associated with CAN diagnosis, but weight loss may in part be modulated by autonomic function not solely attributed to vagal function.

## Ethics Statement

The study was approved by the Scientific Ethical Committee of the Capital Region of Denmark (H-1-2012-031), the Danish Medicines Authority (EudraCT: 2012-001150-26), and the Danish Data Protection Agency. The trial is registered with ClinicalTrials.gov, number NCT01612468.

## Author Contributions

TD, LT, FK, and HA initiated and designed the trial. CH, JF TD, CF, LT, JH FK, SM, and HA participated in data collection and interpretation of data. CH, JF, and DV did the statistical analysis. CH wrote the first draft of the manuscript. All authors have revised the manuscript for crucial intellectual content.

### Conflict of Interest Statement

CF has received research support and lecture fees from Novo Nordisk. JF is consulted and stock owner in Medicus Engineering and received lecture fees from Eli Lilly. DV and LT own stocks in Novo Nordisk, have received lecture fees from and consulted for Merck Sharp & Dome and Novo Nordisk. JH has consulted for Merck Sharp & Dome, Novo Nordisk and Roche. FK has received lecture fees and/or research support from, served on advisory boards of and/or consulted for AstraZeneca, Boehringer Ingelheim Pharmaceuticals, Bristol-Myers Squibb, Eli Lilly, and Company, Gilead Sciences, Merck Sharp & Dohme, Novo Nordisk, Ono Pharmaceuticals, Sanofi and Zealand Pharma. SM has served on advisory boards for Novartis Pharma, Novo Nordisk, Merck Sharp & Dome, Sanofi-Aventis, AstraZeneca, Johnson & Johnson, Roche, Mankind, Boehringer-Ingelheim, Zeeland, Eli Lilly and Intarcia Therapeutics, and has received lecture fees from Novo Nordisk, Merck Sharp & Dome, Astra-Zeneca, Johnson and Johnson, Roche, Shering-Ploug, Sanofi-Aventis, Novartis Pharma, Eli Lilly and Bristol-Meyer Squibb. HA owns stock in Novo Nordisk and has served on advisory boards for Abbott and Novo Nordisk. TD has received research support from Novo Nordisk and AstraZeneca and lecture fees from Novo Nordisk and Boehringer-Ingelheim. The remaining author declares that the research was conducted in the absence of any commercial or financial relationships that could be construed as a potential conflict of interest.

## Abbreviations

30/15 ratio: Heart rate response to standing
ANS: Autonomic nervous system
CAN: Cardiovascular autonomic neuropathy
CARTS: Cardiovascular autonomic reflex tests
E/I ratio: Heart rate response to deep breathing
GLP-1 RA: Glucagon-like peptide-1 receptor agonists
HF: High frequency power
HRV: Heart rate variability
LF: Low frequency power
RMSSD: Root mean square of the sum of the squares of differences between consecutive R–R intervals
SDNN: Standard deviation of normal-to-normal intervals.
